# DNA vaccine encoding heat shock protein 90 protects from murine lupus

**DOI:** 10.1186/s13075-020-02246-4

**Published:** 2020-06-22

**Authors:** Aijing Liu, Fu-Dong Shi, Irun R. Cohen, Giuseppe Castaldo, Giuseppe Matarese, Francisco J. Quintana, Antonio La Cava

**Affiliations:** 1 Department of Medicine, University of California Los Angeles, Los Angeles, CA 90095 USA; 2 Present Address: Second Hospital of Hebei Medical University, Shijiazhuang, China; 3 Barrow Neurological Institute, Phoenix, AZ 85013 USA; 4 Department of Immunology, The Weizmann Institute of Science, 7610001 Rehovot, Israel; 5 Dipartimento di Medicina Molecolare e Biotecnologie Mediche, Federico II University of Naples, Naples, 80131 Italy; 6 Istituto di Endocrinologia e Oncologia Sperimentale, Consiglio Nazionale Delle Ricerche (IEOS-CNR), Naples, 80131 Italy; 7 Ann Romney Center for Neurologic Diseases, Brigham and Women’s Hospital, Harvard Medical School, Boston, MA 02115 USA

**Keywords:** Systemic lupus erythematosus, Heat shock proteins, DNA vaccine, Autoimmunity, T regulatory cells

## Abstract

**Background:**

Systemic lupus erythematosus (SLE) is a chronic autoimmune disease characterized by the presence of autoantibodies to multiple self-antigens, including heat shock proteins (HSP). Because of the increased expression of HSP90 and abnormal immune responses to it in SLE, we investigated whether an HSP90 DNA vaccine could modulate the development and clinical manifestations of SLE in lupus-prone mice.

**Methods:**

(NZB x NZW)F_1_ (NZB/W) mice were vaccinated with DNA constructs encoding HSP90 or control plasmids or vehicle. The mice were then monitored for survival, circulating anti-dsDNA autoantibodies, and immune phenotypes. Renal disease was evaluated by immunohistochemistry and by the measurement of proteinuria.

**Results:**

Vaccination with HSP90 DNA reduced lupus disease manifestations and prolonged the survival of NZB/W mice. The protective effects of the HSP90 DNA vaccine associated with the induction of tolerogenic dendritic cells (DCs) and an expansion of T regulatory cells (Tregs).

**Conclusions:**

The beneficial effects of DNA vaccination with HSP90 in murine SLE support the possibility of HSP90-based therapeutic modalities of intervention in SLE.

## Introduction

Heat shock proteins (HSPs) are evolutionarily conserved proteins that work as molecular chaperones for the intracellular transport and assistance during protein folding, helping in the degradation of unrecoverable denatured proteins [1]. In addition to heat shock, situations of stress and/or pathologic conditions (such as inflammation) also influence the expression and activities of HSPs. For example, an aberrant expression of HSPs has been implicated in the pathogenesis of multiple autoimmune diseases including systemic lupus erythematosus (SLE) [2], where the abnormal expression of HSP90 that correlated with lupus disease activity and autoantibodies also associated with defined polymorphic allelic variants of the *HSP90* gene [3–5]. These findings suggest that a targeted regulation of HSP90 could possibly have effects on the chronic inflammatory response in SLE [6], as also supported by studies of pharmacologic inhibition of HSP90 [7, 8]. However, it remains unclear whether the reported HSP90 abnormalities in SLE represent a cause or a consequence of the disease.

DNA vaccines with plasmids that encode an antigen of interest (whose pathogenic role in the disease has been established) can effectively modulate immune responses to selected antigens in vivo [9]. This strategy has been applied successfully when using plasmids that encode HSPs that are abnormally expressed in autoimmune diseases [10, 11]. Because of this consideration and the fact that immune responses and expression of HSP90 are altered in SLE [3, 12, 13], we decided to investigate whether the use of DNA vaccination—to persistently deliver low-dose HSP90—could influence SLE disease manifestations and clinical outcomes. Our results show that DNA vaccination with HSP90 in lupus-prone (NZB x NZW)F_1_ (NZB/W) mice extended the survival of the animals, decreased the production of anti-dsDNA autoantibodies, and limited renal disease. These protective effects did not occur in mice vaccinated with DNA encoding HSP60 (another HSP that has been associated with other autoimmune diseases but that may apparently not be critical in SLE) [3]. Finally, DNA vaccination with HSP90 induced tolerogenic immune responses that limited the lupus disease manifestations and extended the survival of the lupus mice.

## Methods

### HSP-encoding constructs

Full-length cDNAs of human *HSP60*, *HSP90* genes were cloned individually into the pcDNA3 vector (Invitrogen) under the control of the human CMV promoter (Supplementary Fig. 1A). Briefly, cDNA encoding *HSP60* or *HSP90* in pGEM was amplified using specific oligonucleotides containing restriction sites for *Bam*HI or *Hin*dIII. The amplicon and pcDNA3 vectors were purified and digested with *Bam*HI/*Hin*dIII. The digested PCR product coding for the respective HSP (*HSP60* or *HSP90*) and linearized pcDNA3 vector were ligated using T4 DNA ligase according to standard protocols. The ligated plasmids were used to transform *E. coli* and later sequenced to confirm the correct insertion of the cDNAs. For vaccination, the individual DNA constructs—named pHSP60 and pHSP90 when encoding HSP60 and HSP90, respectively—were prepared in large scale using the EndoFree Plasmid Mega Kit (Qiagen, Santa Clarita, CA). After DNA precipitation with ethanol and resuspension in sterile PBS, endotoxin levels were checked by *Limulus* amebocyte lysate and always found to be under acceptable levels for in vivo use (< 0.02 endotoxin U/μg DNA). Construct quality and purity were reassessed before use by enzymatic digestion, to reconfirm vector map sites.

### HSP90 expression

Translation of HSP90 protein in vitro from pHSP90 was assessed by SDS-PAGE and autoradiography following incubation with ^35^S-methionine (Perkin Elmer, Akron, OH). A 90-kDa protein was detected in the pHSP90 samples, while no ^35^S-labeled product was detected from the control pcDNA3 vector (Supplementary Fig. 1B). The few minor bands detected in the HSP90 preparations were likely degradation products recognized by anti-HSP90 antibodies.

### Mice

(NZB x NZW)F_1_ (NZB/W) mice were purchased from The Jackson Laboratory (Bar Harbor, ME) and maintained at the University of California Los Angeles under pathogen-free conditions. Only female mice were used and treated according to the National Institutes of Health guidelines for the use of experimental animals under protocols approved by the institutional Animal Research Committee. Mice were divided into pHSP90 and pHSP60 groups and two control groups (pcDNA3 and PBS), each containing 10 mice. At 8 weeks of age and 5 days after injection with 50 μl cardiotoxin (10 μM) in the right tibialis anterior muscle, mice were injected in the same area with pHSP60 or pHSP90 constructs or pCDNA3 empty vector (each with 100 μl at 1 μg/μl) or vehicle (100 μl PBS). The mice received a second injection of the same amount of plasmid or vehicle in the left tibialis anterior muscle the following week, having been injected 5 days earlier with cardiotoxin.

### Measurement of antibodies

Blood samples were collected at the beginning of the study and 12 days after the end of the regime of DNA vaccination. Serum was stored at − 20 °C until use. HSP90- and HSP60-specific antibodies were measured by ELISA in flat-bottom microtiter plates precoated overnight with 0.1 μg/well recombinant HSP90 or HSP60 (Boston Biochem, Cambridge, MA) or glutathione S-transferase as control (Sigma Aldrich, St. Louis, MO) in carbonate buffer at 4 °C. Non-specific binding was blocked by incubation with 1% skim milk for 2 h at 37 °C. Serum samples were added diluted 1:100 and incubated for 3 h at 37 °C. Bound IgG were detected using alkaline phosphatase-conjugated goat anti-rat IgG (Jackson ImmunoResearch Laboratories, West Grove, PA) using substrate for alkaline phosphatase (Sigma Aldrich).

### Proteinuria

Albustix reagent strips for urinalysis (Bayer, Pittsburgh, PA) were used to monitor urine protein content. Proteinuria was defined as > 100 mg/dl at two different measurements 1 day apart.

### Elisa

ELISA measured total anti-dsDNA antibodies (Alpha Diagnostic Intl., San Antonio, TX). Subtyping of the anti-dsDNA antibodies by ELISA was done using as secondary antibodies HRP-conjugated anti-IgG1, IgG2a, IgG2b, or IgG3 (eBioscience, San Diego, CA).

### Flow cytometry

Popliteal lymph node cells (draining from the muscle injected with plasmid constructs) were costained for CD11 and MHC class II, CD40, CD80, CD86 using fluorochrome-labeled monoclonal antibodies (mAb). T regulatory cells (Tregs) were costained with fluorochrome-labeled mAb to CD3, CD4, CD25, and Foxp3 (the latter after using the Cytofix/Cytoperm kit, eBioscience). All mAb were from eBioscience. Cells were acquired on a FACSCalibur™ flow cytometer with CellQuest™ software (BD Biosciences, San Jose, CA) and analyzed using FlowJo software (Tree Star, Ashland, OR).

### In vitro assays

Dendritic cells (DCs) from popliteal lymph nodes were isolated on an AutoMACS® separator with the Pan Dendritic Cell Isolation kit (both from Miltenyi Biotec) and cultured in the presence of 10 ng/ml GM-CSF. For extracellular HSP expression, 3 × 10^5^ DCs/well were cultured in 6-well plates for 3 days at 37 °C/5% CO_2_ in the presence of 1.25 μg recombinant HSP90 or vehicle in HL-1 medium (Lonza, Benicia, CA). Cocultures included CD4^+^CD25^−^ T cells isolated as negative fraction with the Mouse CD4^+^CD25^+^ Regulatory T Cell Isolation kit (Miltenyi Biotec). For intracellular HSP90 expression in DCs, Chariot™ transfection reagent (Active Motif, Carlsbad, CA) was used according to the manufacturer’s instructions. Briefly, 1.25 μg recombinant HSP90 was complexed to Chariot™ in 100 μl PBS volume prior to transfection of sorted DCs plated at a concentration of 3 × 10^5^ cells/well in 6-well plates. HSP-transfected DCs were then cultured for 3 days at 37 °C/5% CO_2_ in HL-1 medium with CD4^+^CD25^−^ T cells that had been isolated as negative fraction with the Miltenyi Biotec Mouse CD4^+^CD25^+^ Regulatory T Cell Isolation kit. Intracellular expression of HSP90 in DCs after transfection with Chariot™ reagent was confirmed by western blot 6 h post-transfection of HSP90 in the comparison with non-transfected DCs cultured for 6 h in the presence of the same amount of HSP90 in medium, using anti-HSP90 mAb (BD Biosciences, Franklin Lakes, NJ) followed by detection with ECL Plus reagents (GE Healthcare Chicago, IL).

### Histology

Kidneys from mice of 30–32 weeks of age were embedded in OCT compound, snap frozen, and stored at − 80 °C. For staining, sections of 4 μm were put in cold acetone for 5 min, washed, and blocked with 2% BSA for 1 h before indirect immunofluorescence using FITC-conjugated rabbit anti-mouse IgG (Thermo Fisher Scientific, Waltham, MA). Sections were counterstained with H&E for assessments of glomerular activity score (G.A.S.) and tubulointerstitial activity score (T.I.A.S.) in a blinded manner, using a scale of 0–3, where 0 is no lesions, 1 is lesions in < 30% of glomeruli, 2 is lesions in 30–60% of glomeruli, and 3 is lesions in > 60% of glomeruli. The G.A.S. includes glomerular proliferation, karyorrhexis, fibrinoid necrosis, inflammatory cells, cellular crescents, and hyaline deposits. The T.I.A.S. includes interstitial inflammation, tubular cell necrosis and/or flattening, and epithelial cells or macrophages in the tubular lumen. The raw scores were averaged to obtain a mean score for each feature, and mean scores were summed to obtain an average score from which a composite kidney biopsy score was derived [14].

### Statistical analyses

Differences between individual groups were evaluated using the Student’s *t* test with GraphPad Prism 4 software (San Diego, CA). *P* < 0.05 was considered significant.

## Results

### Immunogenicity of the DNA constructs in vivo

DNA vaccination of NZB/W mice with pHSP90 or pHSP60 associated with circulating anti-HSP90 or anti-HSP60 antibodies, respectively (Supplementary Fig. 2), supporting the functionality of the DNA constructs in vivo.

### DNA vaccination with HSP90 prolongs the survival of NZB/W lupus-prone mice

**Fig. 1:**
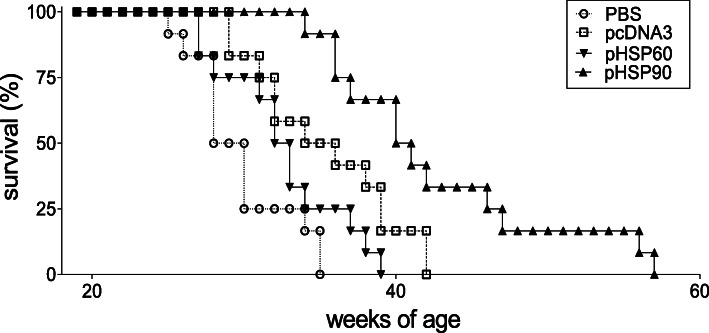
DNA vaccination with HSP90 extends the survival of NZB/W lupus mice. Mice that had been DNA-vaccinated at 8 weeks of age with pHSP60 or pHSP90 or empty pcDNA3 or PBS (*n* = 10 per group) according to the protocol described in the “Methods” section were monitored for survival (*x-*axis). *P* < 0.001 in the comparison between PBS vs. pHSP90 and pcDNA3 vs. pHSP90

Eight-week-old lupus-prone NZB/W mice were DNA-vaccinated with constructs encoding HSP90 or HSP60, according to the protocol described in the “Methods” section. Control mice received an empty construct (pcDNA3) or vehicle (PBS). The monitoring of the animals showed that the mice that had been treated with pHSP90 had a significantly extended survival as compared to all other groups (Fig. 1). There was no significant difference in survival among mice vaccinated with pHSP60 and controls (Fig. 1).

### DNA vaccination with pHSP90 delays lupus disease manifestations in NZB/W mice

**Fig. 2:**
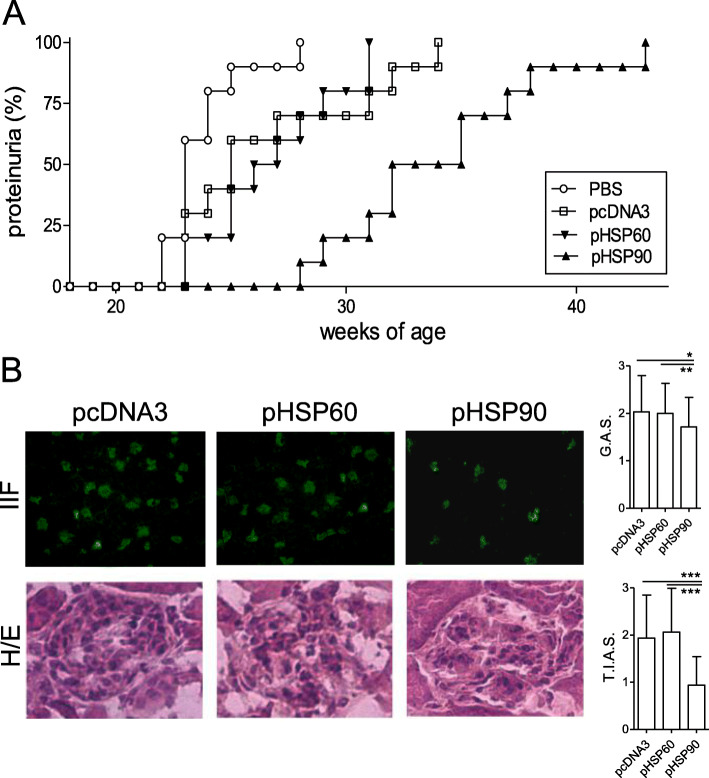
DNA vaccination with HSP90 protects NZB/W mice from lupus disease manifestations. **a** Delayed development of proteinuria in NZB/W lupus mice that had been DNA-vaccinated with HSP90 as compared to HSP60 and controls (*n* = 10 per group). Monitoring occurred at the time points indicated on the *x*-axis. *P* < 0.002 in the comparison between PBS vs. pHSP90 and pcDNA3 vs. pHSP90. **b** Indirect immunofluorescence (IIF) for IgG deposition and hematoxylin/eosin (H/E) staining of renal glomeruli from NZB/W mice that had been DNA-vaccinated with control pcDNA3 or pHSP60 or pHSP90 22 weeks earlier. The graphs show the glomerular activity score (G.A.S.) and tubulointerstitial activity score (T.I.A.S.) measured in a blinded fashion from 6 fields (*n* = 6 mice per group). *P < 0.03 in the comparison between pcDNA3- and pHSP90-treated mice; **P < 0.05 in the comparison between pHSP60- and pHSP90-treated mice; ***P < 0.001 in the comparison between pcDNA3- or pHSP60- and pHSP90-treated mice

**Fig. 3:**
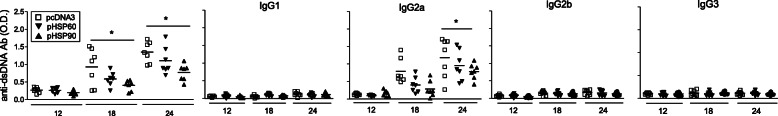
ELISA for anti-dsDNA IgG and their subtypes in NZB/W mice treated with pHSP90, pHSP60, or control vector (pcDNA3) according to the protocol described in the “Methods” section. The *x*-axis indicates the weeks post-DNA vaccination, when sera were analyzed. *P < 0.03 between pcDNA3- and pHSP90-treated mice

Kidney disease is a major clinical manifestation of lupus disease. Monitoring of the DNA-vaccinated mice indicated that none of the pHSP90-treated mice developed proteinuria by the time that all other groups of mice had developed it (Fig. 2a). This delay in the development of renal disease in HSP90 DNA-vaccinated mice associated with histopathological findings of a reduced renal deposition of IgG and lower pathology scores (Fig. 2b), confirming protection from lupus nephritis in pHSP90-vaccinated mice. Additionally, mice that had been DNA-vaccinated with HSP90 had significantly reduced levels of circulating anti-dsDNA autoantibodies, particularly of the subclass associated with Th1 responses (IgG_2a_) (Fig. 3).

### DNA vaccination with HSP90 favors immune regulation

**Fig. 4:**
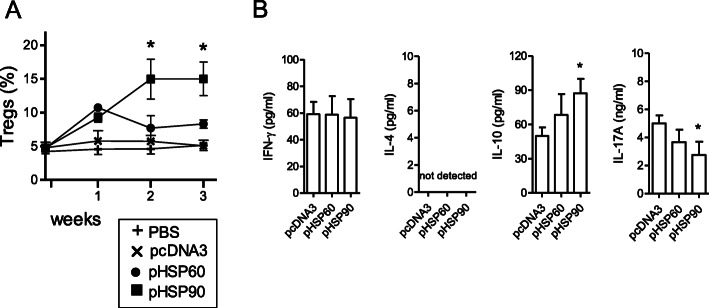
DNA vaccination of NZB/W mice with pHSP90 associates with the induction of tolerogenic immune responses **a** Increased frequency of CD4^+^CD25^+^Foxp3^+^ Tregs in popliteal lymph nodes from mice that had been DNA-vaccinated with pHSP90 as compared to mice receiving pHSP60, empty plasmid (pcDNA3), or PBS. Monitoring was done after DNA vaccination on a weekly basis, ex vivo, by flow cytometry. **b** Concentration of serum cytokine levels at 24 weeks in DNA-vaccinated mice. *P < 0.05 vs. control pcDNA3

Ex vivo flow cytometry analyses on the cells from popliteal lymph nodes—draining the site of DNA injection—showed an increase in the frequency of Tregs in HSP90 DNA-vaccinated mice as compared to all other groups (Fig. 4a). The increased number of Tregs in popliteal lymph nodes in HSP90 DNA-vaccinated mice remained significantly higher as compared to pcDNA3-vaccinated mice up to 24 weeks post-treatment (10.3 + 1.75 vs. 4.2 + 0.5; *P* < 0.03). The increased number of Tregs in HSP90 DNA-vaccinated mice also associated with an increased expression of anti-inflammatory IL-10 and reduced pro-inflammatory IL-17 (Fig. 4b).

### DNA vaccination with HSP90 induces tolerogenic immune responses

**Fig. 5:**
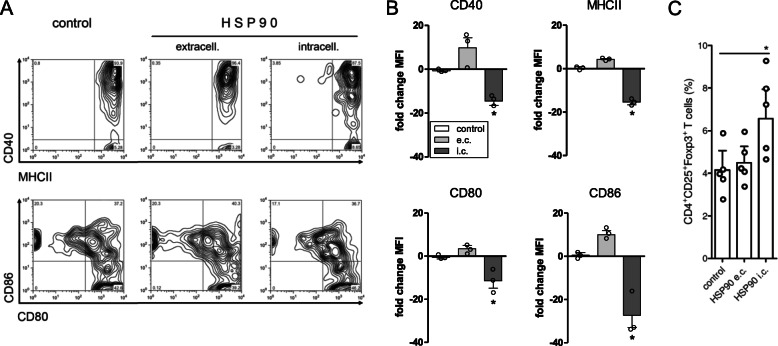
Effects of DNA vaccination with pHSP90 on dendritic cells (DCs). **a** Popliteal lymph node DCs from pHSP90-vaccinated mice (sorted as indicated in Supplementary Fig. 3A) were analyzed for cell surface expression of MHC class II and costimulatory molecules (CD40, CD80, and CD86). **b** Fold changes in median fluorescence intensity (MFI) of the same markers as **a** in DCs where HSP90 was either expressed as intracellular (i.c.) (following transfection with Chariot reagent) or extracellular (e.c.) protein (present in the culture medium of non-transfected DCs). *P < 0.05 in the comparisons with control untreated DCs (0–6 h). **c** Effects of intracellular vs. extracellular HSP90 expression in DCs on Tregs. Reduced expression of MHC class II and costimulatory molecules (CD40, CD80, CD86) in DCs expressing HSP90 intracellularly associated with the expansion of Tregs from CD4^+^CD25^−^ T cells (negatively sorted with magnetic beads) in cultures for 3 days at a 1:4 ratio with DCs. *P < 0.04 between control or e.c. HSP90 and i.c. HSP90. Dots in **b** and **c** represent individual points; columns show cumulative results + SEM

Dendritic cells (DCs) are major players in the induction of Tregs [15]. The activity of DCs is critically influenced by the handling and expression of intracellular antigen as well as by the availability of antigen in the extracellular space. To investigate the potential differential contributions of intracellular vs. extracellular expression of HSP90 in the induction of tolerogenic immune responses, we evaluated in sorted DCs (Supplementary Fig. 3A) the consequences of the expression of HSP90 intracellularly (i.e., secondary to the episomial nature of the encoding construct, Supplementary Fig. 3B) vs. extracellularly (i.e., HSP90 present in the extracellular space [16]). The comparison of the effects of extracellular vs. intracellular expression of HSP90 in DCs showed that intracellular expression of HSP90 in DCs facilitated Tregs induction (Fig. 5). Specifically, larger numbers of Tregs were induced when HSP90 was expressed intracellularly in DCs as compared to extracellularly (Fig. 5c). Consistent with a tolerogenic phenotype [15, 17], a reduced surface expression of MHC class II and costimulatory molecules by flow cytometry was seen on DCs expressing HSP90 intracellularly (Fig. 5).

## Discussion

This study shows that DNA vaccination with HSP90 significantly prolonged the lifespan of lupus-prone NZB/W mice, reduced circulating autoantibodies, and limited renal disease.

HSPs are ubiquitous, phylogenetically conserved molecular chaperones that assist with proper protein folding and stabilization of proteins under conditions of stress, also aiding in protein degradation [1]. Some HSPs are particularly abundant in eukaryotic cells, e.g., HSP90 represents ~ 1–2% of the total cell protein content under physiological conditions [6]. While the most evident role of HSP90 is to help structural maturation and conformational regulation of other proteins (by participating in the assembly of kinases, transcription factors, and steroid receptors), an immunomodulatory role of HSP90 in the spatiotemporal regulation of chaperoned molecules has also been reported [18]. Additionally, the intracellular expression of HSP90 in DCs has been shown to modulate the maturation and antigen presentation of these cells [6–8]. Our results that the intracellular expression of HSP90 in DCs favored an induction of Tregs extend the known cytoprotective effects associated with the expression of HSP90 intracellularly [6]. It remains to be understood whether the contribution of HSP90 to tolerogenicity could be influenced by the complex formation/dissociation of intracellular HSP90 with aryl hydrocarbon receptor [19], a molecule that modulates DC tolerogenicity [20]. Whether or not that may be the case, our findings show for the first time that HSP90 (a modulator of the transport of cargo molecules including antigenic peptides [16] and a provider of danger signals that limit autoantibody pathogenicity [21, 22]) can modulate immune responses in autoimmune conditions. In this context, it has to be noted that distinct HSPs appear to play different roles in the pathogenesis of different autoimmune diseases. DNA vaccination of lupus mice with HSP60 did not confer disease protection, although DNA vaccination with HSP60 could modulate autoimmune diabetes and autoimmune arthritis [23, 24]. Since abnormal HSP60 responses and expression are common in autoimmune diabetes and arthritis [25, 26] but not in SLE [3], it seems reasonable to assume that distinct functions of individual HSPs could have specific roles in the pathogenesis of unrelated autoimmune conditions. In other words, defined HSP activities might provide distinctive physiopathological contributions and/or involvement in the pathogenesis of different autoimmune diseases. For example, effects on autoimmunity would differ if an HSP is important in antigen presentation [6] or rather acts within the mitochondrial matrix [27], where under stressful conditions it can help to correctly fold imported mitochondrial proteins.

To summarize, we found that DNA vaccination with HSP90 extended the lifespan of lupus-prone NZB/W mice by inducing tolerogenic immune responses. Specificity was confirmed by the finding that disease protection was due to the vector-encoded HSP90 product and was absent in control plasmid (pcDNA3)-vaccinated mice, where the plasmid backbone (encompassing immunomodulatory CpG motifs [28]) likely contributed to a small reduction in disease activity (as compared to PBS-treated controls) yet insufficient for a significant protection from SLE.

## Conclusion

In mice that spontaneously develop SLE, DNA vaccination with HSP90 prolonged survival and alleviated disease manifestations by promoting tolerogenic immune responses. These results suggest the possibility of a targeted modulation of HSP90 through DNA vaccination as a new strategy of therapeutic intervention in SLE.

## Supplementary information

**Additional file 1: Fig. S1.** A. Map of the plasmid backbone (pcDNA3) and constructs in which the HSP cDNA had been ligated (between Hind III and BamHI restriction sites) to create either pHSP90 or pHSP60 (see Methods for details). B. pHSP90 translation into HSP90 protein by SDS-PAGE followed by authoradiography. pcDNA3 served as negative control. **Fig. S2.** Immunogenicity of the products of DNA vaccination. IgG antibodies to HSP90 or HSP60 or to control glutathione S-transferase (GST) in vehicle-treated (naïve) or DNA-vaccinated animals (*n* = 8 per group) were assessed by ELISA on sera taken at day 12 after the last injection of the DNA constructs. *P < 0.05 in the comparisons between pcDNA3- and pHSP90 DNA-vaccinated mice. **Fig. S3.** A. Representative gating strategy used to separate DCs as CD11c^+^ cells after pregating for FCS and SSC. B. For the intracellular (i.c.) expression of HSP90, 3 × 10^5^ sorted DCs were transfected with 1.25 μg recombinant HSP90 complexed to Chariot™ reagent. In parallel, the same amount of sorted DCs cultured in the presence HSP90 served as indication of extracellular (e.c.) expression. After 6 h, comparisons were made after washing the cells twice in PBS before western blot with anti-HSP90 antibodies on whole cell lysates.

## Data Availability

The datasets used and/or analyzed during the current study are available from the corresponding author on reasonable request.

## Abbreviations

HSP(s): Heat shock protein(s)
DCs: Dendritic cells
