# Supplementary material for: DNA vaccine encoding heat shock protein 90 protects from murine lupus
Source: Arthritis Res Ther. 2020 Jun 22;22:152. doi: 10.1186/s13075-020-02246-4 (PMC7310240; doi:10.1186/s13075-020-02246-4)
Supplement: Supplementary file 1 — Additional file 1: Fig. S1. A. Map of the plasmid backbone (pcDNA3) and constructs in which the HSP cDNA had been ligated (between Hind III and BamHI restriction sites) to create either pHSP90 or pHSP60 (see Methods for details). B. pHSP90 translation into HSP90 protein by SDS-PAGE followed by authoradiography. pcDNA3 served as negative control. Fig. S2. Immunogenicity of the products of DNA vaccination. IgG antibodies to HSP90 or HSP60 or to control glutathione S-transferase (GST) in vehicle-treated (naïve) or DNA-vaccinated animals (n = 8 per group) were assessed by ELISA on sera taken at day 12 after the last injection of the DNA constructs. *P < 0.05 in the comparisons between pcDNA3- and pHSP90 DNA-vaccinated mice. Fig. S3. A. Representative gating strategy used to separate DCs as CD11c+ cells after pregating for FCS and SSC. B. For the intracellular (i.c.) expression of HSP90, 3 × 10^5^ sorted DCs were transfected with 1.25 μg recombinant HSP90 complexed to Chariot™ reagent. In parallel, the same amount of sorted DCs cultured in the presence HSP90 served as indication of extracellular (e.c.) expression. After 6 h, comparisons were made after washing the cells twice in PBS before western blot with anti-HSP90 antibodies on whole cell lysates. [file 13075_2020_2246_MOESM1_ESM.docx]

**Supplementary Figures**

B

A

pHSP90 pcDNA3

103 kDa

77 kDa

60 kDa

**Supplementary Figure 1.** A. Map of the plasmid backbone (pcDNA3) and constructs in which the HSP cDNA had been ligated (between Hind III and BamHI restriction sites) to create either pHSP90 or pHSP60 (see *Methods* for details). B. pHSP90 translation into HSP90 protein by SDS-PAGE followed by authoradiography. pcDNA3 served as negative control.

**Supplementary Figure 2.** Immunogenicity of the products of DNA vaccination. IgG antibodies to HSP90 or HSP60 or to control glutathione S-transferase (GST) in vehicle-treated (naïve) or DNA-vaccinated animals (n = 8 per group) were assessed by ELISA on sera taken at day 12 after the last injection of the DNA constructs. **P*<0.05 in the comparisons between pcDNA3- and pHSP90 DNA-vaccinated mice.

DCs

i.c. e.c.

B

A

103 kDa

77 kDa

60 kDa

**Supplementary Figure 3.** A. Representative gating strategy used to separate DCs as CD11c^+^ cells after pregating for FCS and SSC. B. For the intracellular (i.c.) expression of HSP90, 3 x 10^5^ sorted DCs were transfected with 1.25 μg recombinant HSP90 complexed to Chariot^™^ reagent. In parallel, the same amount of sorted DCs cultured in the presence HSP90 served as indication of extracellular (e.c.) expression. After 6 hours, comparisons were made after washing the cells twice in PBS before western blot with anti-HSP90 antibodies on whole cell lysates.
